# Heat Shock Proteins Modulate Keloid Formation

**Published:** 2011-04-29

**Authors:** Serhat Totan, Anthony Echo, Eser Yuksel

**Affiliations:** Baylor College of Medicine, Michael E. Debakey Department of Surgery, Division of Plastic Surgery, Houston, TX

## Abstract

**Objective:** Heat shock proteins (HSPs) modulate the intensity of the inflammatory and synthetic response to stress in wound healing. Induction of HSPs at the site of wounds improves healing by acting as a molecular chaperone. However, the role of HSPs may augment the inflammatory response, leading to an uncontrolled synthetic process. Propensity for keloid development involves genetic predisposition, physical factors, and an aggressive inflammatory response. The aim of this study is to demonstrate the differential expressions of HSPs in keloid and normal tissues. **Methods:** Twenty-five keloid and adjacent normal tissue samples were removed from 24 patients who were between 16 and 45 years of age. Western blot, enzyme-linked immunosorbent assay, and immunofluorescence studies were performed to examine hsp27, hsp47, hsp60, hsp70, and hsp90 levels in keloid and normal tissue. **Results:** Our results demonstrated a significant overexpression of hsp27, hsp47, and hsp70 in keloid tissue compared to that of normal tissue. Statistical analysis using the Student *t* test revealed a significant difference between these 2 groups (*P* < .01), while the expression of hsp60 and hsp90 were not significantly different between the keloid and normal tissue samples. **Conclusion:** The overexpression of HSPs indicates that both a proliferative (hsp70) and a matrix synthesis (hsp47, hsp27) component are present in keloid tissue. From this point of view, it is probable that HSPs play a pivotal role in keloid formation. Unveiling HSP-keloid interactions may allow us to manipulate the inflammatory and proliferative phases of wound healing with the potential to control keloid formation.

Keloid scars represent an abnormal healing response in wounded tissue, which can create significant distress for the patient. Keloids are most frequently seen between the first and third decades of life and have a strong correlation with darkly pigment skin, which carries a 15- to 20-fold increased risk for keloid formation.^1^ A variety of skin injuries can result in keloid formation including surgery, traumatic lacerations and abrasions, injections, burns, and any disease causing inflammation in the skin, such as folliculitis or zoster.^2^^-^5 This benign, proliferative disorder is characterized by increased collagen content, as well as increased collagen turnover.^4^^-^7 Because keloids routinely have an inciting traumatic or inflammatory event leading to their formation, the dysregulation of intracellular proteins during the wound healing process likely plays a role in the uncontrolled wound healing response.

The heat shock proteins (HSPs) are probably the most well-studied intracellular molecular chaperones. They are ubiquitous among all living organisms, protecting cells from physiologic stress by stabilizing protein synthesis, transport, and function. The heat shock response was initially described more than 30 years ago in the *Drosophila*, where the upregulation of HSPs were noted.^8^ The expression of HSPs provides a protective cellular response to the encountered stressors. Heat shock proteins also have important regulatory roles in the control of apoptosis; and the regulation of steroid receptors, kinases, and other protein remodeling events.^9^ The conditions found in wounds generate a stressful environment for the cells involved in the repair process. The induction of HSP expression in wounded tissue has been shown to improve healing; however, an altered expression in keloid-prone patients may partially explain their pathogenesis.

Currently, no single therapeutic modality exists that has been shown to be superior in the treatment of keloids, as the complex nature of wound healing and the lack of a proper animal model hinders the development of effective treatment methods. We designed this study with the hypothesis that the overexpression of HSPs in keloid tissue may be responsible for prolonging the extensive inflammatory events that lead to an uncontrolled collagen synthesis. A comparative study utilizing keloid and normal tissue from patient-matched samples was undertaken to evaluate the expression of HSPs to elucidate their roles during the wound healing process.

## METHODS

### General design

This study was approved by the institutional review board for Human Subject Research for Baylor College of Medicine and its affiliated hospitals. Twenty-five keloid samples from 24 patients, who had been scheduled for keloid excision, were included in the study. Keloid tissues were removed by elliptical excision. A part of the keloid sample was sent to pathology for examination and confirmation of the clinical diagnosis. Then the rest of the specimen was processed for the project. Samples were dissected to separate keloid tissue from adjacent normal tissue. After this dissection, keloid and normal tissue samples were processed separately.

### Tissue processing

The keloid and normal tissue samples were minced separately into small 1-mm pieces. Approximately 0.5 cm^3^ of the minced tissue samples were placed into a 50-mL plastic test tube containing 1 mL of radioimmunoprecipitation assay (RIPA) buffer solution. The tissue samples were then homogenized. Each homogenate was centrifuged for 10 minutes to retrieve the supernatant liquid. All protein extracts were normalized to a 1-mg/mL concentration. Enzyme-linked immunosorbent assay (ELISA) and Western blot analyses were performed to determine hsp27, hsp47, hsp60, hsp70, and hsp90 expressions in the keloid and normal tissue samples.

### Western blotting

The gels were soaked in transfer buffer at room temperature, and proteins were electrophoretically transferred to nitrocellulose membranes for 60 minutes. The blots were blocked with 5% nonfat milk in phosphate buffered saline (PBS) solution at room temperature for 60 minutes and subsequently incubated with an anti-HSP antibody (2 µg/mL) at 4°C overnight. After 3 washes with PBS, the secondary antibody was added to the membrane at room temperature for an hour. After intensive washing periods with PBS, positive antigen-antibody binding was detected using luminal reagent. This process was preformed for hsp27, hsp47, hsp60, hsp70, and hsp90.

### Enzyme-linked immunosorbent assay

Protein extract (50 µL of 1-mg/mL solution per well) was incubated overnight at 4°C. The protein solution was then removed, and 200 µL per well of blocking buffer (PBS containing 1% bovine serum albumin (BSA) and 0.02% azide) was placed to block nonspecific protein binding. The plate was washed once with PBS, and 50 µL per well primary antibody (2 µg/mL) was added for 1 hour at room temperature. Then the plate was washed 3 times with PBS containing 0.05% Tween-20. The secondary antibody was added using the same dilution as the primary antibody and incubated for an hour at room temperature. At the end of the incubation period, each plate was washed 3 times with PBS containing 0.05% Tween-20. Illuminol solution was placed into each well, and luminescence was quantitated using a Perkin-Elmar HTS-300 plate reader (Perkin-Elmer, San Jose, CA). This process was performed for hsp27, hsp47, hsp60, hsp70, and hsp90.

### Histology and immunofluorescence

Tissue samples were fixed with 10% Zinc Formalin and stained with Hematoxylin and Eosin (H & E) for tissue histology. Immunofluorescence staining was performed using Texas Red to demonstrate HSP expression on the tissue sections. Cell nuclei were labeled using Hoechst staining.

Immunohistochemical staining of the twenty-five keloid samples and the patient-controlled normal tissue samples were performed following the sectioning of the samples. The tissue sections were rinsed with PBS and then incubated with PBS containing 1.5% normal horse serum for 30 minutes. The mouse antihuman antibody to hsp27 was then incubated for 30 minutes. The tissue sections were rinsed with PBS 3 times for 5 minutes each, and then the Texas Red–labeled horse anti-mouse immunoglobulin G (IgG) was incubated for 30 minutes. The tissue sections were rinsed with PBS 3 times for 5 minutes each. Counterstaining of the cellular nuclei in the tissue sections was performed by incubating the samples in Hoechst solution (1 µg/mL) for 10 minutes. The tissue sections were rinsed with PBS 3 times for 5 minutes each. This process was repeated for hsp47, hsp60, hsp70, and hsp90.

Immunofluorescence was observed with a Nikon E600 fluorescence microscope, and all photographs were taken at the same exposure time. Digitized morphometric analysis was performed to analyze the differential expression of HSPs in normal and keloid tissue slides using ImagePro software (Media Cybernetics, Inc, Bethesda, Maryland).

### Data management

The statistical significance for the expression of each HSP was determined by comparing the results between the keloid and normal tissue groups using the Student *t* test.

## RESULTS

**Figure 1:**
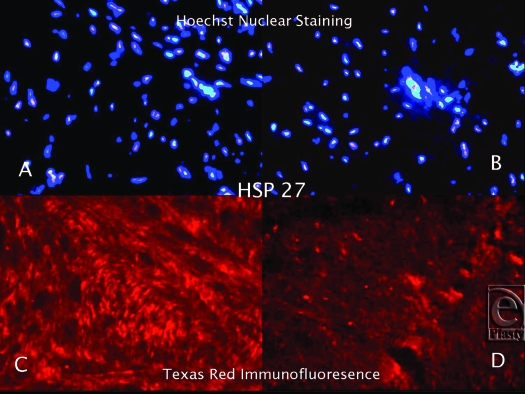
Hoechst Nuclear (*top row*) and Texas Red (*bottom row*) immunofluorescence staining of keloid and normal skin tissues. Double staining was performed in each slide. Images A and C (keloid) are the same microscopic areas in each HSP group which were viewed by different filters. It is also true for Images B and D (normal tissue). Hoechst stain shows the cell population. Texas Red demonstrates hsp27 expression.

**Figure 2:**
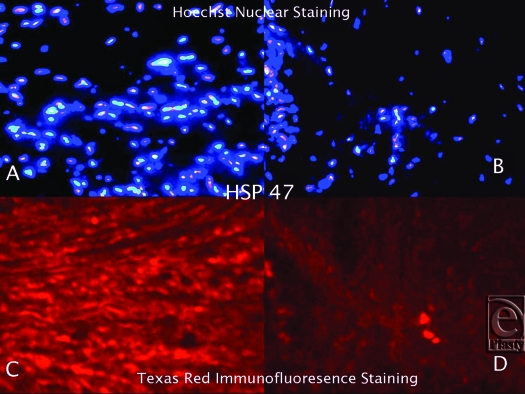
Hoechst Nuclear (*top row*) and Texas Red (*bottom row*) immunofluorescence staining of keloid and normal skin tissues. Double staining was performed in each slide. Images A and C (keloid) are the same microscopic areas in each HSP group which were viewed by different filters. It is also true for Images B and D (normal tissue). Hoechst stain shows the cell population. Texas Red demonstrates hsp47 expression.

**Figure 3:**
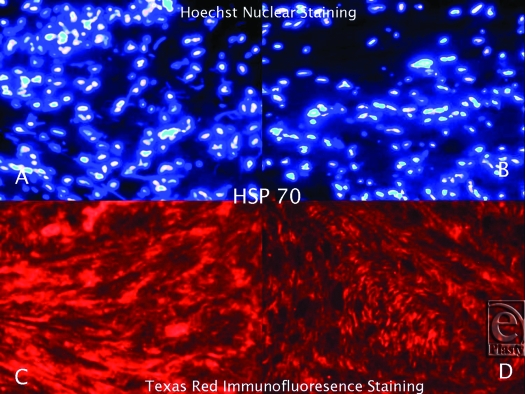
Hoechst Nuclear (*top row*) and Texas Red (*bottom row*) immunofluorescence staining of keloid and normal skin tissues. Double staining was performed in each slide. Images A and C (keloid) are the same microscopic areas in each HSP group which were viewed by different filters. It is also true for Images B and D (normal tissue). Hoechst stain shows the cell population. Texas Red demonstrates hsp70 expression.

Hematoxylin and Eosin staining confirmed that all of the keloid samples had a typical keloid pattern on histological examination. Immunofluorescence staining, utilizing Texas Red, revealed that the keloid tissue samples were strongly bound with anti-hsp27, anti-hsp47, and anti-hsp70 antibodies (Figs 1-3). Although the tissue expression of hsp27, hsp47, and hsp70 was also detected in normal skin, it was significantly lower than the keloid samples. Hsp60 and hsp90 expression in keloid tissues did not differ significantly from the normal skin. Hoechst nuclear staining was also performed to define the cell population in the same HSP expressing tissue regions of each sample. The number of pixels in the HSP-positive areas was calculated, and the ratio of the HSP expression area to the total pixel count in each image was determined. According to these calculations, we demonstrated that tissue expressions of hsp27, hsp47, and hsp70 increased 10-, 16-, and 3-fold, respectively, in keloid tissue when compared to normal skin.

**Figure 4:**
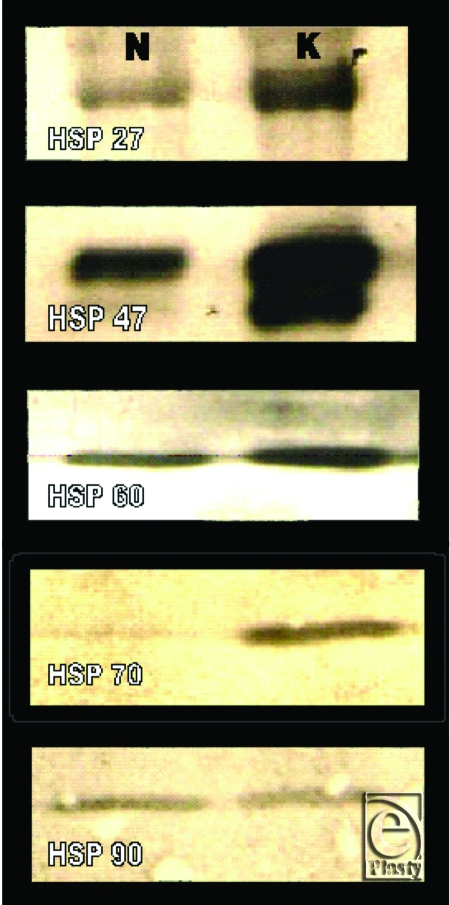
Western blot analysis demonstrating the HSP expression in keloid and normal skin tissue. (N = normal skin, K = keloid).

Western blot and ELISA analyses supported our findings in immunofluorescence microscopy by demonstrating overexpression of hsp27, hsp47, and hsp70 in keloids relative to normal skin (*P* < .01). Tissue expression of hsp60 and hsp90 in keloid tissue was not significantly higher than normal tissue as determined by Western blot (Fig 4) and ELISA analyses (Table 1).

## DISCUSSION

Wound repair is a complex process involving a highly regulated cascade of events requiring coordinated interactions between cells, soluble factors, and extracellular matrix components. Activation of the clotting cascade leads to the release of several vasoactive peptides and chemotactic factors that stimulate inflammatory cell migration. The migrating neutrophils and macrophages cause the release of several growth factors, including platelet derived growth factor, transforming growth factor-β, and insulin-like growth factor-1.^10^,^11^ Ultimately, the transition of an acute wound into granulation tissue requires an equilibrium between the degradation and synthesis of extracellular matrix. Disruption of the equilibrium between these components results in an abnormal healing response. In predisposed individuals, the wound healing process shows overhealing and excessive extracellular matrix production, which can lead to keloid formation.^4^,^6^,^7^

Heat shock proteins are constitutively expressed in tissues under normal conditions, where they act as molecular chaperones; thereby, providing a positive influence on cellular protein configuration and location within the cytosol. They help new or distorted proteins fold into their optimal functional configuration and directly participate in the translocation of proteins from one compartment to another inside the cell. These proteins are also believed to play a role in the presentation of proteins fragments on the cell surface to help the immune system recognize diseased cells. More importantly, during the wound-healing process, they modulate the intensity of the inflammatory and synthetic signal in response to stress.^12^ Delayed and downregulated heat shock response has been demonstrated in people with diabetes, which may contribute to the impaired wound healing in this patient population.^13^

Certain HSPs are rapidly induced in the wound environment, and they play a critical role in the overall process of wound healing.^12^,^14^^-^16 Hsp27, hsp47, hsp60, hsp70, and hsp90 are known to be constitutively expressed in normal healthy skin; however, their expressions are upregulated in stressful conditions as seen in the wound environment.^12^,^13^,^17^^-^19

Hsp27 and hsp47 are closely related to collagen synthesis,^20^,^21^ and their levels of expression are important to elucidate in keloid scars. Tissue expressions of hsp60, hsp70, and hsp90 are closely related to inflammation and inflammatory cytokines;^22^^-^26 therefore, they were also investigated in this study.

### Heat shock protein 27

Heat shock protein 27, in several studies, has been declared as a marker of cellular differentiation and is found in stratum spinosum and stratum granulosum of normal skin or in the suprabasal cell layers of hyperproliferating epidermis.^27^^-^30 Interestingly, its expression is decreased in basal and squamous cell carcinomas,^31^ which are known to present as nonhealing skin ulcers. In wounded tissue, hsp27 is known to regulate endothelial cell migration by affecting the formation and stabilization of actin microfilaments.^32^^-^34 It modulates actin filament dynamics in response to various stimuli through mitogen-activated protein (MAP) kinases.^35^ Through this process, it also regulates fibroblast adhesion, elongation, and migration, causing increased wound contraction.^36^ The wound contraction process is controlled through the extracellular signal-regulated kinases (ERK) and p38 kinases cascades,^35^,^37^ where mechanical stress increases hsp27 expression through the phosphorylation of p38.^38^ A recent study demonstrated that the inhibition of hsp27 phosphorylation lead to a decreased expression of connective tissue growth factor and collagen type I production in keloid fibroblasts.^20^

### Heat shock protein 47

Located in the endoplasmic reticulum of collagen-producing cells, hsp47 acts as a collagen-specific molecular chaperone during the biosynthesis and secretion of procollagen. It specifically regulates collagen processing and the formation of collagen type I and III during wound healing.^21^,^39^ The formation of the triple-helical structure is an important posttranslational event in collagen synthesis.^40^ In the endoplasmic reticulum, hsp47 binds to the alpha polypeptide chains to align the formation of the triple helix, then dissociates from the procollagen molecule, and then enters into Golgi apparatus.^17^,^19^,^22^,^41^^-^44 Hsp47 is a good marker of proper wound healing as an indicator of collagen biosynthesis. Elevated levels of hsp47 are associated with increased collagen production, which contributes to scar formation and wound strength. Prolonged expression is associated with disease states such as a Dupuytren's contracture, which is known for its proliferation of fibroblasts and collagen.^45^ Naitoh et al^46^ demonstrated upregulation of hsp47 in keloid tissues, and our results revealed similar findings regarding hsp47 expression. Other studies have demonstrated that suppression of hsp47 decreased scar formation and wound strength.^47^,^48^ Although these results suggest that hsp47 is one of the possible triggering factors in keloid formation, its role seems to be limited to the collagen synthesis phase and fails to explain the inflammatory response in wound healing, which plays an important role in keloid pathogenesis.

### Heat shock protein 60

Heat shock protein 60 predominantly facilitates the folding and assembly of proteins within the mitochondrial matrix and stabilizes preexisting proteins under stressful conditions.^49^,^50^ During wound healing, hsp60 is elevated in the basal and low suprabasal cells of the epidermis.^9^,^51^ Both endogenous^52^ and exogenous^53^ hsp60 expression during the inflammatory process has shown to increase epithelial cell migration; however, with aging its response appears to diminish.^54^ Unfortunately, the expression of this regulatory protein was not found to be differentially regulated in the keloid samples from our study.

### Heat shock protein 70

The heat shock protein 70 family comprises an abundant and highly conserved group of molecular chaperones. Heat shock protein 70 functions by binding to the hydrophobic side chains of nonnative peptides in extended conformations and folds them into their native state. Heat shock protein 70 prevents protein aggregation by shielding the hydrophobic sites on the new synthesized, unfold polypeptides.^55^ In times of stress, this process inhibits apoptosis by increasing the cells ability to deal with elevated concentrations of unfolded or denatured proteins.^49^ However, decreased cellular levels of hsp70 was associated with delayed wound healing in diabetics,^56^,^57^ hypercortisolic states,^58^ and the elderly people.^59^ In several studies, a strong correlation between wound healing and an upregulation of hsp70 has been emphasized,^60^^-^62 which is correlated with greater tensile strength in the healing wound.^63^ Positive correlations were observed between the serum levels of hsp70 and various markers of inflammation, such as IL-6, TNF-α, and IL-10.^25^ Our results supported these findings that because the healing response during keloid formation is aggressive, an upregulation of hsp70 is expected.

### Heat shock protein 90

Heat shock protein 90, an abundant cytosolic protein, acts as a molecular chaperone in vitro that promotes refolding of denatured proteins, holds denatured proteins in a folding-competent state for other chaperones, and prevents protein unfolding and aggregation.^17^,^64^^-^66 This study has demonstrated that hsp90 expression in tissues is not significantly different between keloids and normal skin. Although hsp90 is present in skin, it is not found in large amounts compared to other tissues.^12^ Therefore, it may not have an important regulatory function in wound healing.

This study demonstrated that the expression of hsp27, hsp47, and hsp70 is upregulated in keloid tissue. This overexpression of HSPs may cause an overactive response in the healing parameters during wound healing, leading to keloid formation. However, the exact localization of these regulatory proteins in the molecular pathway has yet to be elucidated. The Rho kinase signaling cascade may have a regulatory role in the modulation of keloid formation through HSPs.

### Future directions

Future studies for understanding the role of HSPs in keloid formation will look in to the rho kinase signaling cascade. Rho is a small GTPase in the rho family, which is involved in many aspects of cell behavior, such as motility, proliferation, size regulation, centrosome positioning, and apoptosis.^67^ It has also been demonstrated that the signaling pathway underlying the effects of Prostaglandin E2 acts through Rho activation.^68^ Segain's The experiment of Segain et al^69^ presented more evidence in support of this enzyme's role with inflammation, in which he demonstrated that a Rho kinase blockade prevents inflammation via nuclear factor-κB (NF-κB) inhibition. Murata et al^70^ demonstrated that Rho kinase inhibition has a significant therapeutic effect on established liver fibrosis in rats. These findings demonstrate the role of the Rho kinase system in inflammation and fibrosis, which are important processes during wound healing and more specifically keloid formation. Recently, several studies have correlated the Rho kinase system to HSPs. Wang and Bitar^71^ demonstrated that inhibition of Rho A leads to the disappearance of hsp27 distribution within the cell, which suggests that Rho A may exert its effect on cytoskeletal reorganization via hsp27. Another study demonstrated that inhibitors of 3-hydroxy-3-methylglutaryl CoA reductase (statins) upregulate endothelial nitric oxide synthase (eNOS) by inhibition of Rho GTPase.^72^ It has also been shown that binding of hsp90 to eNOS enzyme results in its rapid activation and nitric oxide release.^72^ However, to date, there is no scientific data to clearly define the role of Rho kinases in skin wound healing and scar formation. More studies will need to be performed before a clear connection between Rho kinase and keloid formation can be determined.

## CONCLUSION

In this study, we demonstrated the differential expression of HSPs in keloid samples and normal tissues samples with patient-matched controls, specifically the upregulation of hsp27, hsp47, and hsp70. This correlates with the pathophysiological development of a keloid, which demonstrates increased collagen (hsp27, hsp47) and inflammation (hsp70). It was surprising that the hsp60 and hsp90 expressions in the keloid tissue samples were not significantly different than the basal levels in the normal skin. Their roles as molecular chaperones in the mitochondria (hsp60) and cytosol (hsp90) may not be as critical for keloid formation.

The close functional relationship between HSPs and Rho kinases may further support our aforementioned hypothesis, which needs to be further investigated.

## Figures and Tables

**Table 1:** The expression of the different heat shock proteins in keloid and normal tissue samples (*n* = 25). The two samples were compared with the Student t test

	Normal Tissue (mg/mL)	Keloid Scar (mg/mL)	*P*
hsp27	12478 (SD = 3495)	18598 (SD = 2643)	<.01*
hsp47	4707 (SD = 831)	6874 (SD = 1641)	<.01*
hsp60	5015 (SD = 1158)	5316 (SD = 978)	.11
hsp70	4645 (SD = 864)	6566 (SD = 1047)	<.01*
hsp90	4597 (SD = 937)	4799 (SD = 1038)	.07

*Statistically significant.
